# Mastoiditis and facial paralysis as initial manifestations of Wegener's Granulomatosis

**DOI:** 10.1590/S1808-86942012000200013

**Published:** 2015-10-20

**Authors:** André Souza de Albuquerque Maranhão, Vitor Guo Chen, Bruno Almeida Antunes Rossini, José Ricardo Gurgel Testa, Norma de Oliveira Penido

**Affiliations:** a MD, ENT (Student at the Otorhinolaryngology and Head and Neck Surgery Department's Master's Program at UNIFESP-EPM); b Resident Physician (Resident Physician in the Department of Otorhinolaryngology and Head and Neck Surgery at UNIFESP-EPM); c MD, ENT (Otology Fellow at the Department of Otorhinolaryngology and Head and Neck Surgery at UNIFESP-EPM); d PhD in Medicine (Adjunct Professor in the Department of Otorhinolaryngology and Head and Neck Surgery at UNIFESP-EPM); e Post-doctoral degree in Otorhinolaryngology (Professor in the Department of Otorhinolaryngology and Head and Neck Surgery at UNIFESP-EPM). Universidade Federal de São Paulo (UNIFESP)-Escola Paulista de Medicina (EPM). Hospital São Paulo. Hospital 9 de Julho

**Keywords:** facial paralysis, mastoiditis, wegener granulomatosis

## Abstract

Wegener's Granulomatosis (WG) is characterized by necrotizing granulomas and vasculitis. If left untreated, the prognosis is poor – a 90% mortality rate within 2 years. Several authors have described the otologic manifestations of WG; these authors, however, have not mentioned the stage of the disease in which these findings present – whether as initial manifestations or subsequent to other findings.

**Aim:**

To describe three confirmed cases of WG with mastoiditis as the first manifestation, progressing to peripheral facial paralysis (PFP).

**Material and Method:**

A clinical series study. Patients diagnosed with WG that initially presented with otologic findings are described.

**Results:**

The three cases presented with unilateral otalgia, otorrhea, and hearing loss associated with ipsilateral PFP. None recovered in spite of the treatment; an investigation of associated diseases was therefore undertaken. Positive ANCA-C titers where detected in all patients, confirming the diagnosis of WG. Clinical improvement was seen after treatment of WG; the PFP regressed and hearing thresholds improved partially.

**Conclusion:**

Complications of otitis media (mastoiditis and PFP) that do not respond to the usual treatment require an investigation of associated diseases; WG should be included for an early diagnosis to change the prognosis in these patients.

## INTRODUCTION

Wegener's granulomatosis is an idiopathic systemic form of vasculitis characterized by the presence of necrotizing granulomas and vasculitis in the upper airways, lower airways and kidneys; however, as it is a systemic disease, it may involve any organ. Etiology is yet undefined, but it appears to be a disease of autoimmune nature^1^, ^2^. As it is a rare disease, its actual incidence cannot be precisely determined^1^. It is estimated that prevalence ratios revolve around 3:100.000 inhabitants, with males and females being equally involved. Incidence peaks between the ages of 20 and 40 years^2^. Prognosis is poor, as the disease presently has no treatment. Ninety percent of the patients die within two years. Nonetheless, if therapy is offered early on (before kidney injury onset) with immunosuppressants, long term remission is reached for 90% of the patients^1^.

ENT manifestations are present in the vast majority of patients (73–99%), and are usually among the first symptoms to set in. Therefore, ENT physicians have a determining role in recognizing the early onset of the disease and starting the proper therapy. Occasionally, ear conditions are the first and only to appear^3^. They are present in 20–61% of the cases^4^, otitis media with effusion being the most common manifestation^2^. Various authors cite ear manifestations in the progress of Wegener's granulomatosis; however, they fail to specify when such manifestations set in as part of the disease, i.e., either early on or subsequently to other findings^3^.

This paper aims to offer an update on the possible early isolated clinical manifestations of ear injury connected to Wegener's granulomatosis. The cases of three patients diagnosed with Wegener's granulomatosis are described, showing mastoiditis at first and then peripheral facial nerve palsy. The patients' clinical and audiometric progress is also discussed, alongside the time needed to reach a firm diagnosis.

## MATERIALS AND METHODS

This stud comprises a series of cases f patients diagnosed with Wegener's granulomatosis with early ear manifestations. The results of lab, audiometry, and imaging tests were analyzed, as well as patient clinical progress. This study was submitted and approved by the institution's Ethics Committee and given permit number CEP 0081/10.

## RESULTS

### Case 1

**Figure 1:**
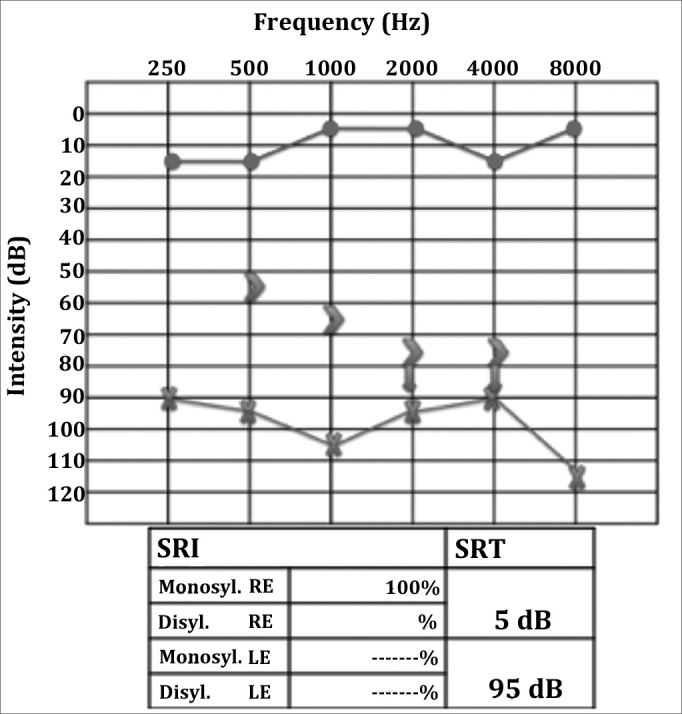
Left ear profound mixed hearing loss; PB max could not be verified.

RCTD, 52, female, was having intense left ear otalgia and rhinorrhea for 20 days. She had been unsuccessfully treated with amoxicillin and had her pain improved after taking intramuscular ceftriaxone. However, she developed left peripheral facial nerve palsy and came to be seen at our service. Her facial nerve palsy was categorized as grade III (House-Brackmann) and otoscopic examination showed a perforated tympanic membrane with effusion. The rest of her test was normal. She was diagnosed with acute otitis media and peripheral facial nerve palsy. CBC, audiometry, and temporal bone CT scans were ordered. The patient was prescribed ciprofloxacin and oral steroids. Audiometry tests showed moderate to severe mixed hearing loss on the left ear and CT scans revealed opacification of the mastoid air cells and the tympanic cavity, with preserved bone septations consistent with acute otomastoiditis. Effusion had ceased and facial nerve palsy had almost entirely regressed two weeks into therapy (the patient still had minor lower lip asymmetry). Otoscopic examination showed bilaterally preserved tympanic membranes but a thickened left eardrum. Control audiometry revealed worsened hearing thresholds (Figure 1) and profound mixed hearing loss in the left ear. An MRI of the inner ear was ordered.

**Figure 2:**
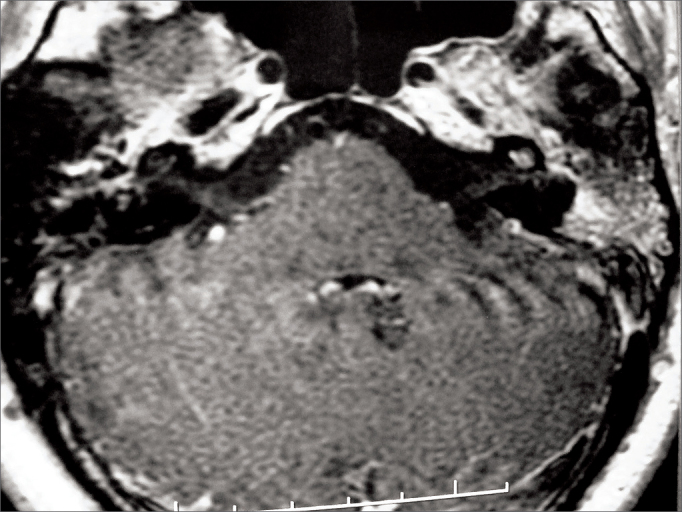
T1-weighed head MRI scan, axial view after contrast has been injected at the level of the inner ear canal; intense contrast uptake seen in the left ear's cochlea and mastoid cells.

**Figure 3:**
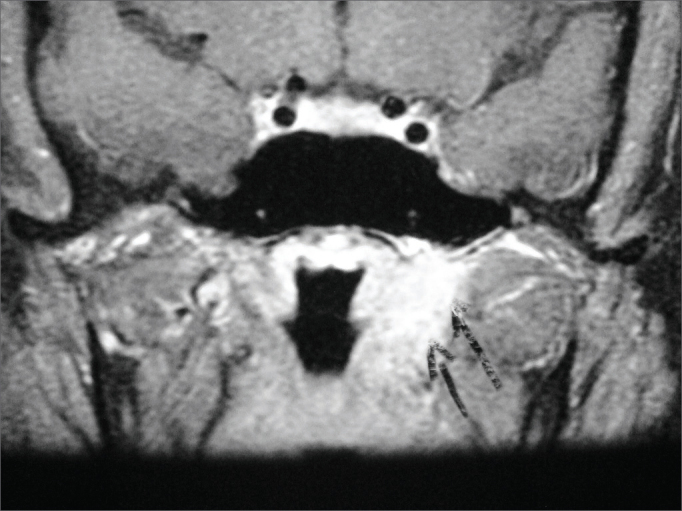
T1-weighed head MRI scan, coronal view after contrast injection; expansive injury seen on the left side wall f the rhinopharynx consistent with reactive lymphnode. The arrows indicate shifting of adjacent parapharyngeal fat.

**Figure 4:**
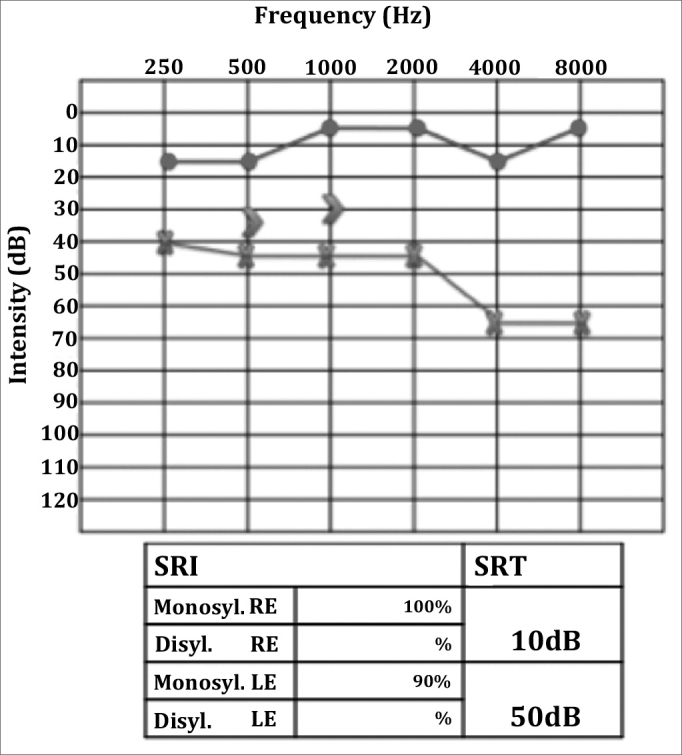
Audiometry after therapy. Left ear mild to moderate mixed hearing loss, with marked improvement on speech discrimination.

In the next visit the patient complained or hearing loss in the right ear. Her MRI scans showed marked contrast enhancement in the cochlea and in the left facial nerve, ipsilateral mastoid opacification, (Figure 2), and a left parapharyngeal lymphnode (Figure 3). Audiometry then revealed right ear conductive hearing loss. The patient was hospitalized for etiology investigation. During her stay the patient had fever episodes. Her CBC was negative, her CSF was normal and free of infection, and her ESR was elevated (140). The patient was analyzed for infectious and rheumatic disease and was suspected for granuloma. His c-ANCA was titrated at 1/160, confirming the diagnosis of Wegener's granulomatosis three months after the onset of symptoms. Chest CT scans revealed the presence of lung parenchymal nodules. Therapy with deflazacort and cyclophosphamide was initiated, and the patient partially recovered her hearing thresholds (Figure 4), as inflammation in the middle ear was controlled.

### Case 2

**Figure 5:**
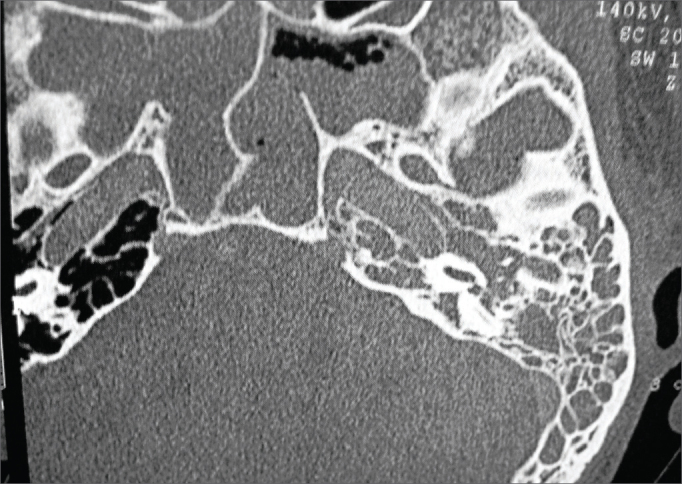
Temporal bone CT scan, axial view showing matter with soft tissue density on the mastoid and in the left middle ear and sphenoidal sinus.

JCM, 31, male, was having otalgia, hypacusis and effusion on the left eat for one month. One week after the commencement of symptoms, he presented right nasal obstruction with hyaline rhinorrhea. The patient looked for care at another service and was diagnosed with acute rhinosinusitis and treated with antibiotics (he could not tell which) for 14 days with no improvement. He started coughing insistently and decided to look for our service. Left year otoscopic examination revealed a reddish thickened eardrum with effusion in the outer ear canal, but no perforation was seen. The patient was treated with cefuroxime axetil and topical ciprofloxacin on his left ear. He came back three days later with peripheral facial palsy (House-Brackmann's grade IV). Temporal bone CT scans revealed opacification involving the left middle ear and mastoid cells without signs of osteolytic injury (Figure 5), the sphenoid sinus bilaterally, the right maxillary and ethmoid sinuses, and matter with soft tissue density in the right nasal cavity. Endoscopic examination showed only a yellowish secretion in the right middle meatus. The patient was diagnosed with acute otomastoiditis with peripheral facial nerve palsy. He was hospitalized and treated with ceftriaxone, clindamycin, and dexamethasone. His CBC suggested leukocytosis (14000) without shifting to the left; eosinophilia, kidney function, ESR, electrolytes, and coagulation were normal; audiometry revealed moderate left ear mixed hearing loss. The patient was discharged after spending six days in hospital and was sent home with a prescription for antibiotics.

**Figure 6:**
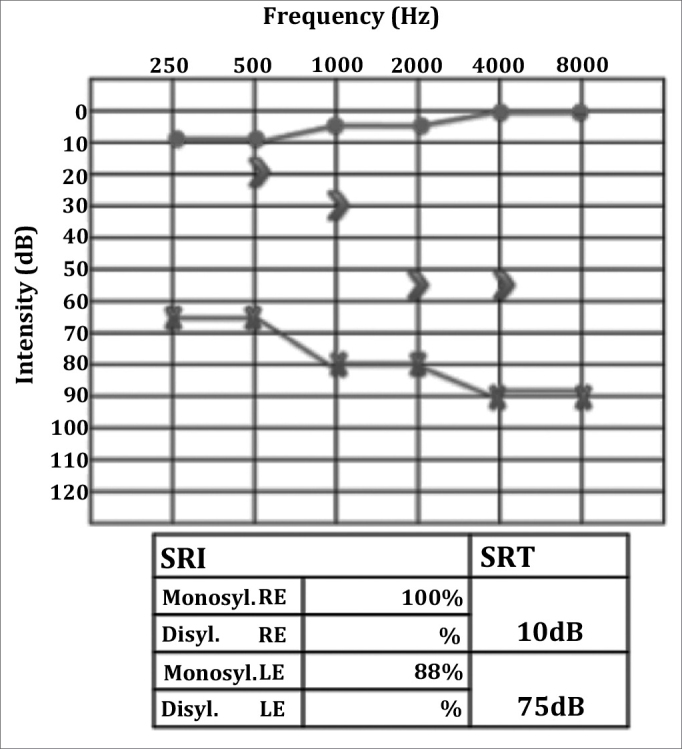
Left ear severe mixed hearing loss.

**Figure 7:**
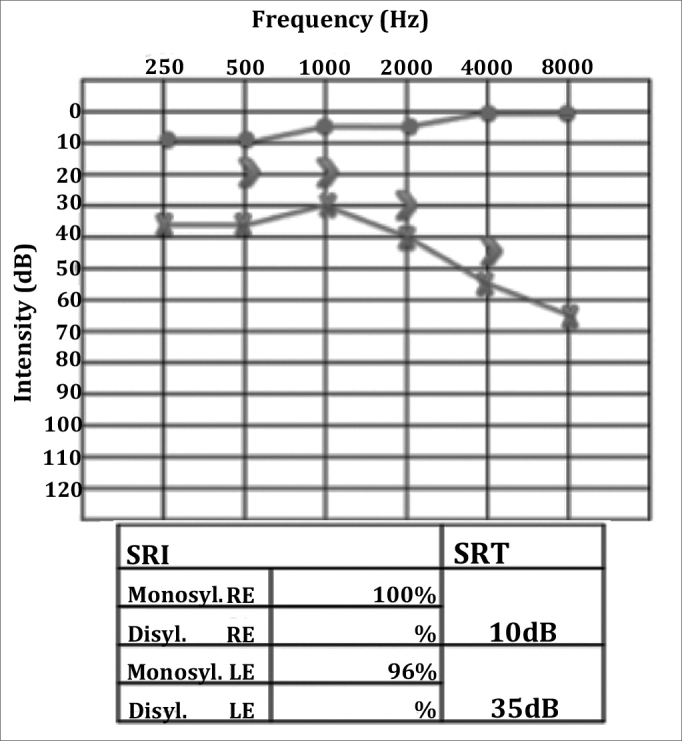
Left ear mild to moderate mixed hearing loss.

In his follow-up visit the patient complained of otalgia, vertigo episodes, and worsening hearing loss. Audiometry showed his tone thresholds had deteriorated (Figure 6). A tympanomastoidectomy was performed on the patient's left ear and the lesions found in the right nasal cavity were biopsied. During surgery we could see mastoid cells filled with some whitish hard material and brittle bone tissue. Biopsies were done on the right nasal cavity. Pathology tests suggested chronic inflammatory process with presence of eosinophils. Temporal bone osteomyelitis was considered, and after discussing the case with the institution's infectologist, we opted to initiate therapy with cefepime. While the patient was hospitalized, serology tests for HIV, hepatitis B and C, VDRL, rheumatoid factor, c-ANCA, and p-ANCA. All came back negative. The rheumatologist was once again called to assess the patient and ordered a c-ANCA test that this time came back positive, with a titration of 1/20. The patient was prescribed cyclophosphamide 100mg/day and prednisone 70mg/day. Chest CT scans showed multiple parenchymal lung nodules. Diagnosis as established 74 days after symptom onset. The patient recovered completely from facial nerve palsy and improved his hearing thresholds only partially (Figure 7).

### Case 3

**Figure 8:**
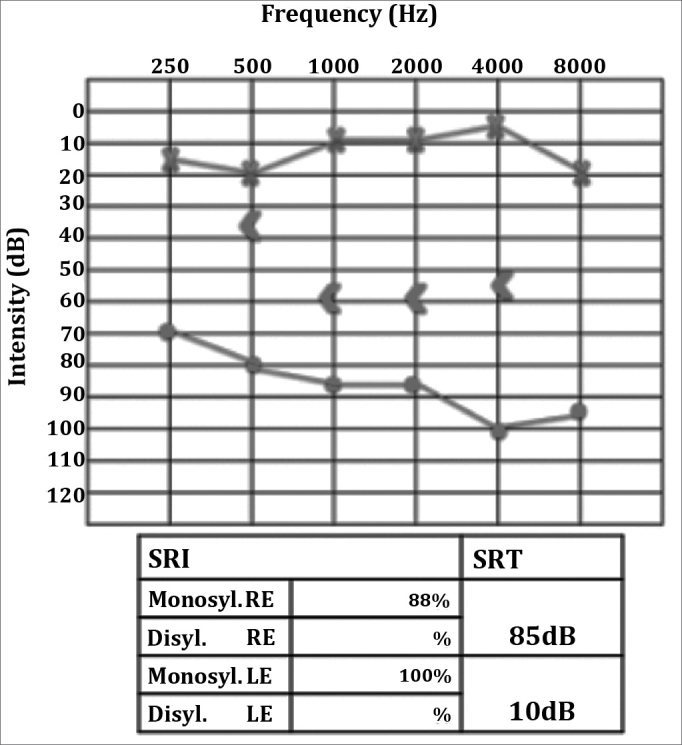
Right ear severe to profound mixed hearing loss.

A Caucasian female patient aged 42 was having intense otalgia and purulent effusion in her right ear for 45 days. She started treatment for otitis media with effusion with antibiotics (amoxicillin-clavulanic acid and ciprofloxacin) and both systemic and topical steroids, but her condition worsened, as hypacusis, vertigo and headaches set in. Twenty days later she was hospitalized and given antibiotics (ceftriaxone) and intravenous steroids. However, the broad spectrum antibiotics and steroids led to no improvement in her condition. As part of her history, she had an unidentified renal disorder and a sister who had undergone kidney transplantation. Otoscopic examination revealed a central perforation on her right eardrum, on which a reddish polypoid tumor and purulent effusion could be observed. The remainder of her ENT tests were normal. Audiometry showed right ear mixed dysacusis (Figure 8). Temporal bone CT and head MRI scans showed complete opacification of the mastoid and the right middle ear associated with otomastoiditis.

Effusion culture findings revealed growing pneumococcus sensitive to the antibiotics used to treat her; CBC showed neutrophilia and shift to the left, elevated ESR, and proteinuria. As the patient did not respond to drug therapy, she was referred to tympanomastoidectomy and placement of a ventilation tube to the right to try and resolve the inflammatory process and collect material for more culturing and histology studies. Seven days after surgery the patient presented progressive peripheral facial nerve palsy (her electroneurogram showed very low potentials and complete nerve degeneration), left ear hearing loss, dyspnea when physically strained, and bloody rhinorrhea. Nasal endoscopy revealed ulcerated bilateral lesions covered by fibrin on the torus tubarius and on the side wall of the right nasal cavity. X-rays and chest CT scans showed the presence of infiltrate and nodules on both lungs.

Pathology tests run on collected mastoid specimens indicated the presence of granulomas.

**Figure 9:**
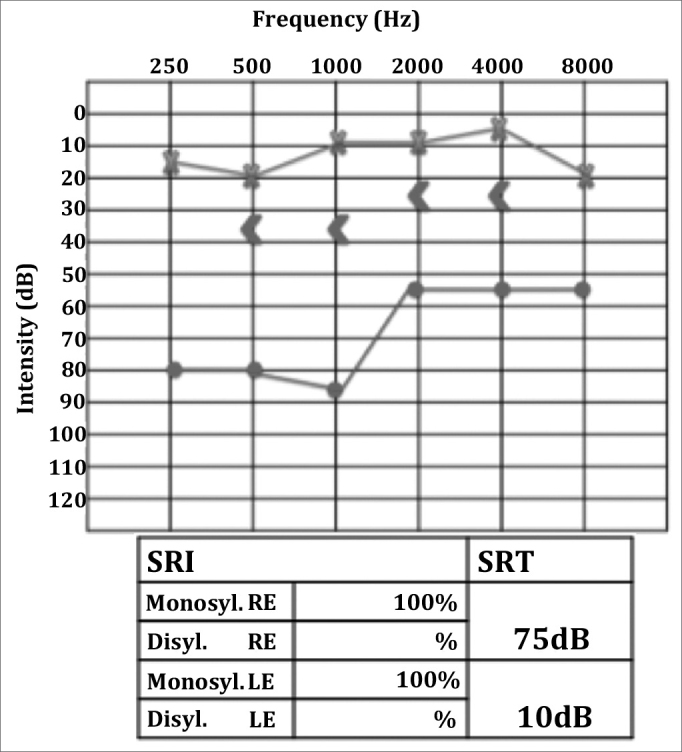
Right ear moderate to severe mixed hearing loss, with improved speech discrimination.

C-ANCA was positive to titrations of up to 1/160, thus confirming the diagnosis of Wegener's granulomatosis two months after symptom onset. The patient was prescribed deflazacort and cyclophosphamide and improved significantly from her systemic involvement and recovered gradually from both the facial nerve palsy and the hearing impairment (Figure 9).

## DISCUSSION

Wegener's granulomatosis is a relatively rare condition^4^. Godman & Churg^5^ have established the criteria to diagnose the disease: 1) granuloma in upper airways; 2) necrotizing vasculitis; 3) glomerulonephritis. Limited forms of the disease were described later by other authors^6^, ^7^. Most of them presented head and neck involvement. The nose and the paranasal sinuses are the most frequently involved sites and account for as many as 90% of the cases.

Ear disorders are observed in 20–61% of the cases^4^, and rarely are the first and only manifestation^7^. These disorders have been categorized as follows: 1) otitis media with effusion (OME); 2) chronic otitis media (COM); 3) sensorineural dysacusis (SND)^8^. Most patients with Wegener's granulomatosis accompanied by ear involvement have otitis media with effusion caused by eustachian tube disorders. Primary involvement of the nasal cavity and paranasal sinuses with nasopharyngeal inflammation have been proposed to account for the eustachian tube disorders seen in these patients, who usually respond poorly to OME drug therapy and undergo the placement of ventilation tubes^7^, as also seen in the cases presented herein. We therefore stress that in cases of persisting OME in adult patients with no history of eustachian tube disorder the ENT physician must thoroughly examine the nasopharynx before placing ventilation tubes. Chronic otitis media occurs consequently to the direct involvement of the middle ear and the mastoid cavity by necrotizing granuloma^9^, and may develop accompanied by effusion, mastoiditis, and facial nerve palsy. Otomastoiditis associated with facial nerve palsy is seen in 10% of the cases^2^, ^4^. In our case base all patients evolved this way, without however presenting other previous symptoms, as reported few times in the literature^3^, ^7^, ^9^.

Several authors recommend that such complications be managed conservatively only, as the nerve function is recovered in most cases, thus challenging the efficacy of surgery^2^, ^4^, ^7^. Nonetheless, when such complications are the first manifestations of the disease, early diagnosis is extremely difficult to produce. Therefore, we believe that many patients with Wegener's granulomatosis will still undergo mastoidectomy, mainly in limited forms of the disease in which the degree of clinical suspicion is even lower as also argued by Dagum et al.^9^. The surgical findings seen in cases 2 and 3 described in this study (obliteration of mastoid cells by granulation tissue) are very similar to those reported by other authors^3^, ^9^. One study reported that a patient with post-mastoidectomy facial nerve palsy recovered after he was treated with immunosuppressants^4^, as also seen in our case 3.

The SND mode of action in patients with Wegener's granulomatosis is yet unclear. Some have postulated that the vasa nervorum and the cochlear vessels are affected by vasculitis, or that immune complexes deposit on the labyrinth to trigger true immune-mediated labyrinthitis, or even that they are adversely affected by the toxic effects of inflammation byproducts arising from the middle ear through the round window^4^, ^9^, ^10^. One should stress that these patients rarely show vestibular symptoms such as vertigo and nystagmus, and that sensorineural hearing loss usually sets in gradually within days to weeks^10^. This pattern of hearing loss was seen in all three cases described in this paper, and served as a warning sign that led us to keep on investigating the condition's etiology. The presence of serum eosinophilia and eosinophils in the pathology tests on case 2 pointed to Churg-Strauss syndrome, a condition characteristically accompanied by peripheral eosinophilia and eosinophilic tissue infiltrate^11^. However, after the c-ANCA titration came back positive, the diagnosis of Wegener's granulomatosis was confirmed.

Wegener's granulomatosis must be considered when patients do not improve as expected despite being given adequate treatment, when they have unspecific systemic symptoms suggesting systemic disease (fever, myalgia, arthralgia), or when other organs are involved (eyes, kidneys, lungs and others). Prolonged evolution times of over 20 days to observe regression of ear inflammation suggests a specific etiology to support disease activity. Histopathological analysis of biopsy material is an important diagnostic tool; the following are usually found: small vessel vasculitis, granuloma, necrosis, and microabscesses^12^. However, one can rarely obtain a large enough sample and multiple procedures are required, as biopsies are inconclusive in over 50% of the times. The paranasal sinuses are the preferred biopsy sites^2^. The determination of serum levels of cytoplasmic antineutrophil cytoplasmic antibodies (c-ANCA) revolutionized the diagnosis of Wegener's, boasting specificity of 99%. A positive test result practically confirms the diagnosis. Sensitivity depends on how active and extensive the disease is, and values can get to as high as 93% during the condition's active phase^8^. Limited manifestations have a high rate of false negatives (30%). However, when there is suspicion, it is recommended to perform a series of titrations^2^.

Wegener's granulomatosis is a lethal disease if left untreated. Therapy consists of steroids and immunosuppressants such as cyclophosphamide, azathioprine, and methotrexate. Long term remission is achieved in up to 90% of the cases, especially in the absence of kidney injury^1^. Controversy still looms over the use of trimethoprim/sulfamethoxazole to induce remission in refractory patients and to maintain remission^1^.

## CONCLUSIONS

Complications from acute otitis media (mastoiditis and peripheral facial nerve palsy) refractory to usual drug therapy require the investigation of granulomatous diseases. Wegener's granulomatosis must be considered so that early diagnosis is made possible and a better prognosis is offered to those affected by this disease.
